# Improving Public Health Through Access to and Utilization of Medication Assisted Treatment

**DOI:** 10.3390/ijerph8104102

**Published:** 2011-10-24

**Authors:** Thomas F. Kresina, Robert Lubran

**Affiliations:** Division of Pharmacologic Therapies, Center for Substance Abuse Treatment, Substance Abuse and Mental Health Services Administration, 1 Choke Cherry Road, Rockville, MD 20857, USA; E-Mail: rlubran@samhsa.gov

**Keywords:** medication assisted treatment, medical care, infectious diseases, opioid abuse, opioid dependence, recovery

## Abstract

Providing access to and utilization of medication assisted treatment (MAT) for the treatment of opioid abuse and dependence provides an important opportunity to improve public health. Access to health services comprising MAT in the community is fundamental to achieve broad service coverage. The type and placement of the health services comprising MAT and integration with primary medical care including human immunodeficiency virus (HIV) prevention, care and treatment services are optimal for addressing both substance abuse and co-occurring infectious diseases. As an HIV prevention intervention, integrated (same medical record for HIV services and MAT services) MAT with HIV prevention, care and treatment programs provides the best “one stop shopping” approach for health service utilization. Alternatively, MAT, medical and HIV services can be separately managed but co-located to allow convenient utilization of primary care, MAT and HIV services. A third approach is coordinated care and treatment, where primary care, MAT and HIV services are provided at distinct locations and case managers, peer facilitators, or others promote direct service utilization at the various locations. Developing a continuum of care for patients with opioid dependence throughout the stages MAT enhances the public health and Recovery from opioid dependence. As a stigmatized and medical disenfranchised population with multiple medical, psychological and social needs, people who inject drugs and are opioid dependent have difficulty accessing services and navigating medical systems of coordinated care. MAT programs that offer comprehensive services and medical care options can best contribute to improving the health of these individuals thereby enhancing the health of the community.

## 1. Introduction

Drug abuse and dependence is a growing public health problem [1]. Most significant is the global increase in opioid abuse and dependence [2]. Opioid abuse and dependence is increasing because of the availability of opioids through increased global trafficking of heroin and the widespread increase in use of opioid analgesics in the treatment of chronic non cancer pain as well as in acute pain management [2,3]. Opioids are highly addictive compounds and individuals at high risk who chronically use opioids, either as an illicit drug or in the management of pain, can develop a substance use disorder.

Substance use disorders are defined in the Diagnostic and Statistical Manual of Mental Disorders, Fourth Edition [4] as substance abuse and substance dependence. Opioid abuse and dependence are chronic, relapsing diseases that can be successfully medically treated. However, they are complex physiologic, social, and behavioral disorders that often coexist with psychiatric illness, as well as, co-morbid medical infectious diseases such as the HIV, hepatitis virus infection or tuberculosis [5–7]. Thus, opioid abuse and dependence are most effectively treated through a set of comprehensive medical, social, psychological and rehabilitative services that address all the needs of the individual [7,8]. MAT, the use of effective medications in combination with behavioral therapies, is a highly effective treatment for opioid abuse and dependence [9]. MAT impacts public health through the reduction of opioid use, opioid overdose mortality and transmission of infectious diseases [10].

## 2. Opioid Abuse and Dependence and Injection Drug Use

Exposure to addictive substances is widespread, but vulnerability to substance abuse and dependence is behaviorally complex, being a function of biological, psychological and environmental interactions and influences [11–14]. Although opioid dependence is characterized by an increased quantity/frequency of use, the clinical diagnosis of opioid abuse and dependence is based on maladaptive patterns of opioid use, cognitive, behavioral, and physiological symptoms as well as significant consequences related to opioid use [4]. The characteristic of tolerance to opioids based on continued use is particularly important in the context of the transition from non-injection opioid use to injection opioid use.

Drug users transition from non-injecting drug use to injection drug use because injecting drug use is considerably more cost effective and provides a more intense drug effect compared to non-injection of drugs [15–17]. In addition, studies have shown that the transition to injecting drug use is more likely for individuals who consume drugs at higher frequencies. There are also social aspects in the transition to injection drug use [18]. Drug users who are homeless or who have been physically abused as well as those with social ties to injection drug users are more likely to transition to injecting. The influence of social networks on drug use practices is thought to occur both directly and indirectly in the context of social comparison and perceived norms that increase risk factors. Opioid injection drug users socially connect with other injectors and participate in long term injecting social networks that reinforce injecting drug behaviors. These opioid social networks tend to comprise a small number of members who develop long term relationships with each other.

The transition from non-injection drug use to injection drug use presents a formidable public health risk for the transmission of both HIV and hepatitis virus infections [19]. The progression to injection drug use results in an unhealthy lifestyle with a poor quality of life, increased morbidity and an estimated 12–20 higher risk of mortality [20–22]. The elevated morbidity and mortality results from a disenfranchisement from medical care, a behavior that produces a high risk of infections, medical complications, and chronic diseases due to unsafe injection practices and sexual risk taking [23–25]

However, injection drug users are not solely at–risk. Non-injection drug users are at-increased risk of death due to drug overdose, and sexually transmitted diseases, including HIV [26,27]. In cities with substantial harm reduction and drug treatment programs, the prevalence of HIV infection has been shown to be virtually identical for injection drug users and non-injection drug users whether or not they are in drug treatment [28]. In this case, sexual HIV risk has become the predominant transmission route for HIV infection [29]. Prescription opioid abusers have also been shown to be at risk for HIV infection due to an increased number of sexual partners [29]. Prescription opioid abusers have been shown to have a high prevalence of psychiatric co-morbidities compared to non-opioid drug users [30]. Thus, both injecting and non-injecting drug users exhibit unhealthy lifestyles with the need for substance abuse treatment that comprehensively addresses their medical, social, and psychological needs.

## 3. Medication Assisted Treatment for Opioid Dependence

In the United States, the use of pharmacotherapies in combination with counseling and behavior therapies to provide a comprehensive therapeutic approach to the treatment of opioid abuse and dependence is termed “Medication Assisted Treatment” or MAT. Federally supported research studies have developed and shown that the most efficacious treatments for opioid abuse and dependence comprise MAT and include psychosocial counseling, financial, legal, educational services as well as wrap around social services [8]. Federal programs catalogue such evidence-based best medical practices and promote their implementation in the care and treatment of patients to optimize good medical outcomes through the education and training of these medical practices by health care providers [16]. Federal programs can also support the piloting of treatment improvement projects to develop national implementation strategies. Drug treatment programs that utilize MAT are regulated in the US by the federal government in their adherence to treatment standards through accreditation and in their record keeping requirements for use of controlled pharmaceuticals [31].

Currently, the US Food and Drug Administration (FDA) has approved four medications for the use in the treatment of opioid abuse and dependence (see Table 1). Methadone is a Schedule II opioid agonist and has demonstrated safety and efficacy in the treatment of heroin abuse and dependence for over forty years and more recently in the treatment of prescription opioid abuse and dependence [32,33]. However, in the United States methadone is only available in specialized treatment programs, called Opioid Treatment Programs (OTPs), that are regulated by Center for Substance Abuse Treatment (CSAT), Substance Abuse and Mental Health Services Administration (SAMHSA). Primary care providers, therefore, are unable to provide this treatment to patients, but can refer patients needing methadone maintenance treatment to an OTP, if available in their region. SAMHSA has recently published a state-by-state profile of OTPs for 2008 that is updated yearly [34].

Buprenorphine is a Schedule III partial opioid agonist which allows for qualified and specially licensed physicians to treat patients with opioid addiction in a primary care setting. The Division of Pharmacologic Therapies at CSAT/SAMHSA provides an online physician and treatment program locator at: http://buprenorphine.samhsa.gov/bwns_locator/. In addition, CSAT/SAMHSA provides a state-by-state profile of the locations of physicians with waivers to prescribe buprenorphine. Buprenorphine treatment of opioid abuse and dependence is reviewed in Treatment Improvement Protocol 40 [35]. Buprenorphine has been shown to be safe and effective in the treatment of both injection (heroin) opioid use and prescription drug abuse and dependence [36]. Buprenorphine has also been developed in a combination form with naloxone to reduce the risk of diversion for illicit use. Models to implement buprenorphine in HIV clinical settings have also been piloted [37]. The HIV/AIDS Bureau of the Health Resources and Services Administration (HRSA) has developed and supported a Special Project of National Significance entitled “*The Buprenorphine Initiative: An Evaluation of Innovative Methods for Integrating Buprenorphine Opioid Abuse Treatment in HIV Primary Care*”. This five year initiative, which began in September 2004, comprises 10 demonstration sites coordinated by a technical assistance/evaluation center that works collaboratively to refine planned interventions, addresses state-of-the-art treatment and policy issues relating to the use of buprenorphine opioid abuse treatment in HIV primary care settings, and conducts local and multi-site evaluations and disseminate findings. A special issue of the *Journal of the Acquired Immune Deficiency Syndrome* (JAIDS) has been published providing a comprehensive analysis of the projects [38].

Naltrexone is a non-narcotic long-acting, opioid antagonist that blocks opioids binding to the mu opioid receptor. Unlike methadone, there is no negative reinforcement (opioid withdrawal) upon discontinuation. Due to naltrexone’s opioid antagonism, patients must abstain from opioids for a minimum of seven days prior to starting treatment to avoid the precipitation of opioid withdrawal. The effectiveness of naltrexone treatment depends upon patient motivation and social support system [39]. Thus, in cultures where there is strong family or social support for the patient in care, oral naltrexone has been shown to be effective in the prevention of relapse to heroin use [40]. Because of a lack of positive reinforcing effects with naltrexone and low motivation on the part of many patients, as well as, poor clinician acceptability, oral naltrexone is not widely prescribed for the treatment of opioid dependence in the United States and thus not seen as a good maintenance treatment medication for relapse prevention.

Vivitrol is an injectable extended-release naltrexone preparation that has recently been approved for the treatment of opioid abuse and dependence. Vivitrol addresses the concern of medication adherence as a monthly injectable formulation and this extended formulation has been shown to be more effective than oral naltrexone [41]. This was also shown in a recent Phase 3 clinical trial that confirmed vivitrol’s safety and efficacy in the prevention of relapse to heroin use in a cohort of injection drug users. A higher retention in care and higher rates of opioid-free urine screens were observed along with a significant reduction in opioid craving compared to placebo. Currently, studies are underway to determine the most efficacious service model(s) for the use of vivitrol in the treatment of relapse prevention to heroin use.

## 4. Health Service Models for Medication Assisted Treatment (MAT)

Health service programs deliver MAT in a regulatory environment where both the federal government and state/local government provide a regulatory framework for the access to and delivery of medications that are controlled by international convention [42]. In the United States, state and local regulations can enhance the federal regulations, but they cannot replace the federal regulations. The MAT federal regulations can be found in the Code of Federal Regulations [43] and establish procedures to determine if a health practitioner is qualified to dispense methadone in the treatment of opioid abuse and dependence in opioid treatment programs, as well as, the quantity of methadone that can be provided for unsupervised use by patients. Thus, the federal regulations address the balance needed in the use of controlled medications for treatment versus the restrictions to limit diversion of the addictive medication.

The MAT federal regulations regulate health service models restricting the dispensing of methadone to OTPs, but allows buprenorphine to be provided in an office base setting by registered and trained physicians. There are no prescribing restrictions for naltrexone and vivitrol, since these medications are not scheduled narcotics with federal controls. Federal regulations that allow the use of MAT in office based settings enhance access to MAT by providing additional venues where patients access pharmacotherapy for opioid dependence. The differing regulations for the use of methadone and buprenorphine in the treatment of opioid dependence result in differing treatment locations and treatment environments. There are a limited number of OTPs in the United States that dispense both methadone and buprenorphine, while there are only three OTPs that dispense exclusively buprenorphine. The highly regulated OTP where methadone is predominately used to treat opioid dependence are considered ‘strict” treatment environments with required daily appearance for medication and mandated urine tests for illicit drug use [43]. Office based opioid treatment (OBOT), on the other hand, was developed, in part, to reduce the stigma and discrimination associated with the treatment of opioid dependence. Providing pharmacotherapies for opioid dependence to patients in a medical office setting is less structured than an OTP and based on the 2005 evaluation of OBOT has resulted in the following: approximately one-third of patients new to substance abuse treatment; roughly sixty percent new to pharmacotherapy; less than ten percent switching from methadone to buprenorphine treatment ; and a majority of patient noted to be receiving treatment for dependence to prescription drug (non-heroin) opioids [44].

The treatment environment and range of services provided either in OTPs or in OBOT are important in maintaining patients in care and treatment. Treatment programs that are patient centered, provide a supportive environment and meet the needs of the patient, regardless of the pharmacotherapy used in treatment, result in patients remaining in treatment for longer periods of time. Patients who remain in treatment have better treatment outcomes due to adherence compliance and persistence [45–47]. These are important characteristics in designing model MAT programs to ensure good clinical and public health outcomes. Barrier free access to MAT is important to obtain maximal public health impact and reach all opioid abusing individuals seeking treatment [48]. Thus, MAT programs providing comprehensive services as part of the continuum of care (see Table 2) in an enabling environment result in quality and effective substance abuse treatment services that promote individual well-being and improved community health.

Medical treatment programs that provide MAT as part of a comprehensive set of services can interface substance abuse treatment services with primary medical care, infectious disease services, and social/rehabilitation services. That interface can be comprehensive through the integration of substance abuse treatment services, primary medical care, infectious disease prevention, care and treatment and social/rehabilitation services. An integrated care and treatment model where MAT services are provided within primary care uses a single medical record minimizes the stigma and discrimination associated with drug treatment services while improving overall health outcomes, particularly for patients with multiple health issues, in a cost-effective manner [50].

For OTPs, methadone dispensing, primary medical care, infectious disease services, and social/rehabilitation services are integrated on-site in the structured environment where methadone is dispensed [51,52]. Based on patient needs, various types of health services can be integrated into OTP MAT services including primary care, mental health, and infectious diseases. Some of the limiting factors for the integration of services are the organizational structure of the OTP, incorporating new procedures in primary care and cost of services [52,53].

### 4.1. Integrated Models of MAT

Buprenorphine, an opioid agonist medication, is approved and regulated in the United States for OBOT. As noted earlier, an office based setting provides enhanced access to buprenorphine in a primary care environment, where health services can be integrated [54]. Multiple models have been piloted for the integration of MAT using buprenorphine within HIV primary care [55]. These include an on-site combination of addiction treatment/HIV specialist treatment; a HIV primary care physician prescribing buprenorphine; a non-physician health care provider integrating medical care and substance abuse treatment services using buprenorphine; and a community outreach model where buprenorphine is provided along with medical services in a mobile van [55]. These pilot projects have uncovered barriers to integrating MAT using buprenorphine within HIV primary care that are both financial and regulatory. Regulatory challenges include licensing and training restrictions imposed by the Drug Addiction Treatment Act of 2000 and confidentiality regulations for alcohol and drug treatment records [56]. A recent study has shown that in a primary care setting using buprenorphine, prescription opioid dependent patients showed better clinical outcomes compared to patients who were dependent on heroin [57].

Naltrexone is a non-narcotic and therefore non-controlled medication for the treatment of opioid abuse as well as alcohol abuse. Naltrexone integrated with mental health services, particularly psychosocial treatment has been shown to be an effective maintenance treatment for reducing heroin use after detoxification [58]. In addition, using clonidine and naltrexone together can be successfully integrated into a primary care setting [59]. In this study retention in care and successful detoxification from opioid abuse was observed with MAT using either naltrexone or buprenorphine. In other care settings, treatment of alcohol use disorders using naltrexone has been successfully integrated into the treatment of patients who have tuberculosis [60]. Current efforts are determining the optimum conditions to integrate vivitrol (extended release naltrexone) into HIV primary care programs. Additionally, how to integrate vivitrol into an OTP setting and in primary medical care as a relapse prevention intervention for patients following buprenorphine detoxification, respectively is currently moving forward[61].

### 4.2. Coordinated Care Models of MAT

Medical service programs can connect substance abuse treatment services with primary medical care and social/rehabilitation services in a non-integrated but coordinated fashion. In these programs, MAT services work with primary medical care and social/rehabilitation services to provide good patient outcomes and enhance community health. MAT, health services and social/rehabilitation services can be separately managed with a different network of health care providers but co-located to allow convenient utilization of primary care, MAT and other services. An additional coordinated approach provides primary care, MAT and other services at distinct locations through differing network of health care providers. A recent study has shown twice as many patients retained in MAT when the MAT services were provided at single location compared to referral of MAT to a distant location [62]. However, coordinated programs can be effective when case managers, peer facilitators, or care navigators promote or support service utilization at the various locations. For example a referral system was modeled with linkage to treatment services for substance use, mental health and social services for HIV+ patients receiving HIV primary care [63]. Patients in this model were referred to MAT either at an OTP or in an office based setting using buprenorphine. An alternative model provided highly stable OTP patients with a 28 day supply of methadone doses and required a monthly check-in. Successful patients were noted to have increased family and social activities and failed patients were provided stepped treatment intensification [64].

A unique Induction/Maintenance model for buprenorphine is the model where a substance abuse treatment specialist provides the initial treatment (induction) with buprenorphine until the patient is stabilized. Then the patient is transferred/referred to a primary care physician who then provides maintenance buprenorphine treatment and medical primary care. This so called ‘wheel and spoke model’ allows for substance abuse treatment specialists to manage the more difficult portion of buprenorphine treatment (early treatment –or induction phase) while the primary care medical program manages the long term maintenance phase of buprenorphine treatment [65]. This model is important in the United States since the Drug Addiction Treatment Act of 2000 limits the number of patients a qualified buprenorphine treatment provider can manage in their practice [66]. This model has been adapted to HIV+ patients where the buprenorphine induction is performed by the substance abuse treatment specialist and then the patient is transferred/referred to the HIV primary care physician [67].

Coordinated MAT for patients seeking relapse prevention interventions after detoxification for opioid can be provided by naltrexone or the recently approved longer acting, extended release vivitrol. As noted earlier, naltrexone it is not widely prescribed for the treatment of opioid dependence in the United States, but is provided as an office based treatment for opioid dependence after detoxification. In addition, studies have shown that the extended release formulations are effective in reducing opioid use and retaining patients in care after detoxification [68,69].

## 5. Public Health and Recovery from Opioid Dependence

The stages or phase of MAT are shown in Table 3. The patient travels through these three stages of treatment, sometimes linearly and sometimes oscillating between phases. The ultimate goal of entering MAT is a good clinical outcome which includes the recovery from opioid abuse and dependence and social reintegration back into society. The individual in recovery is a functioning member of the community and contributes to the public health of the community. Thus, the foundation that MAT is structured upon obtaining recovery from opioid abuse and dependence [70].

The recovery process is the way in which a person actively manages their substance use disorder with efforts to reclaim a full and meaningful life in the community. Recovery is a personal process of growth and change which embraces hope, autonomy and the elements that results in re-establishing a satisfying and productive life. MAT becomes a recovery oriented system of care when its services and integration with other medical, social and rehabilitative services support the individual’s and family’s long term efforts to reclaim full and meaningful lives in the community. Important in recovery is the provision of comprehensive services in the context of MAT but also a supportive, enabling environment that fosters individual responsibility over one’s health and empowerment to change to a healthy lifestyle [71]. Promoting the change to a healthy lifestyle is the development of an individual risk reduction program to reduce the risk of newly acquired sexually transmitted infection and complicating co-morbidities.

MAT as a recovery orientated system of care has four phases, as shown in Table 4, along with a set of recovery oriented strategies and services [72]. An important consideration in phase four, long term sustained recovery, is the personal and clinical decision to continue with medical maintenance of pharmacotherapy or to taper the medication. In either case, the home or living environment is critical to the prevention of relapse to opioid use. To prevent relapse of opioid use the dependent needs a drug free environment. While significant gains have been made through national prevention programs such as “Drug Free Communities”, it remains a Herculean task to keep a community entirely free of illicit drug use. Thus, for long term recovery the home or living environment is where recovery is nucleated [73]. Local peer recovery programs and recovery oriented systems of care that link to or provide individualized, quality long term care that supports recovery from a diagnosis of opioid dependence are critical [74]. These settings provide a network of people to support abstinence and a low risk environment to support recovery. Receiving abstinence support, guidance and information from a recovery home that is committed to long term sobriety reduces the risk of relapse to illicit opioid use. These homes need to be considered as a fundamental component in the development and maintenance of the public health of communities.

## Conclusions

The use of pharmacotherapies in the treatment of opioid dependence in the form of medication assisted treatment is an important tool to enhance public health. Integrating the substance abuse treatment services that comprise medication assisted treatment with other medical, social and community services provides the best platform for promoting recovery from opioid dependence.

## Figures and Tables

**Table 1:** Approved pharmacotherapies comprising medication assisted treatment for opioid abuse and dependence.

*Methadone*—federally regulated through OTP; long-acting opioid receptor agonist for pharmacological therapy; can be used in detoxification or in long term (maintenance) treatment
*Buprenorphine*—office-based opioid treatment or OTPs; federally regulated, long-acting opioid receptor partial agonist for pharmacological therapy; can be used in detoxification or in long term (maintenance) treatment
*Naltrexone*—office-based and substance abuse treatment programs; non-narcotic opioid receptor antagonist for relapse prevention without abuse liability or reinforcing effects; used after detoxification from opioids to prevent relapse to opioid use
*Vivitrol*—extended release naltrexone; non-narcotic opioid receptor antagonist for relapse prevention without abuse liability or reinforcing effects; used after detoxification from opioids to prevent relapse to opioid use

**Table 2:** Elements of the continuum of care in the treatment of opioid abuse and dependence [49].

(1) Prevention of drug initiation

Interventions to reduce the risk of drug and alcohol initiation of use and abuse
Interventions to reduce the risk of sexually transmitted diseases, including HIV
Individual targeted interventions through the life span
Family targeted interventions
Community interventions

(2) Identification of substance use conditions

Screening for drug use-patient self-report, prescription monitoring programs, collaterals
Case finding
Assessment and diagnosis

(3) Initiation and engagement in drug treatment

Brief intervention
Promoting engagement, case management/care navigators
Detoxification/Withdrawal management
Assessment of social, co-morbid medical conditions and co-occurring disorders
Pharmacotherapy

(4) Long term treatment of substance use illness

Psychosocial
Treatment of co-morbid medical conditions and co-occurring disorders
Promote social stability through addressing legal, social, educational, financial issues

(5) Primary care and post treatment management of patient

Recovery
Relapse prevention
Rehabilitation
Medical home

**Table 3:** Stages or phases of MAT.

Induction
○ Medication is chosen based on clinical and patient circumstances—addiction history, severity of withdrawal symptoms, available social support and overdose diagnostic severity ○ MAT initiation where initial dosing of medication is observed and dosing titration is performed by a clinician ○ Dosing and dose titration is based on expression and control of withdrawal symptoms and is a critical period in terms of risk of opioid overdose in treatment ○ Procedures for patient observation during and after dose titration are incorporated into the clinic setting ○ Induction can last 7–10 days with the goal of obtaining a therapeutic dose of opioid medication
Stabilization
○ Stabilization phase occurs when the patient no longer exhibits drug seeking behavior or craving ○ The correct dosage of medication is critical (overdosing versus underdosing) as well as successful participation of the patient in behavioral therapies and rehabilitation services ○ MAT provider determines stabilization based on patient symptoms, not on opioid free urine samples ○ Individual patient health (e.g., pregnancy, liver disease, *etc*.), other medical treatments including HAART and TB treatments, and other drug use or alcohol consumption affects stabilization and is addressed ○ Individual risk assessments are performed and risk reduction interventions implemented to reduce the risk of co-infections and co-morbidities
Maintenance
○ Maintenance pharmacotherapy occurs when the patient is responding optimally to medication treatment and routine dose adjustments are not needed. ○ Patients at this stage have stopped using illicit opioid and resumed productive lifestyles away from the local drug culture. ○ It is also at this stage that patients should have minimal or normal medical needs and can move away from intensive drug treatment settings and receive their medications in a primary care/community setting. ○ Typically take-home medication is allowed for patients ○ If maintenance phase cannot be reached, other drug dependence treatment approaches should be explored to complement MAT

**Table 4:** Phases and goals of MAT recovery oriented systems of care.

Recovery initiation and stabilization
○ Major goal—introduce and educate the patient on pharmacotherapy; eliminate use of illicit opioid use as well as other drugs of abuse for at least twenty-four hours ▪ Educate the patient about the risk and benefits of pharmacotherapy ▪ Provide a choice of alternate/supplemental therapeutic approaches ▪ Identify patient’s treatment needs and engage ▪ Monitor sedative and side effects of medication ▪ Asses safety and adequacy of each dose after administration ▪ Discourage self-medication of withdrawal symptoms ▪ Assess and initially address medical, social, legal, family and other problems including risk reduction strategies ▪ Develop initial coping and craving strategies
Early recovery and rehabilitation
○ Major goal—empower individuals to cope with life problems, medical needs co-occurring disorders vocational and educational needs, family problems, legal issues and develop long term goals for education, employment and family reconciliation ▪ Insure medication dose promotes daily comfort ▪ Link patient to family and peer-recovery support ▪ Develop recovery plan ▪ Assess and address personal strengths and needs
Recovery maintenance
○ Major goal—patient assumes primary responsibility for their life ▪ Patient receives needed integrated services ▪ Patient is active in community recovery support programs ▪ Patient receives take home medication from an OTP ▪ Decision on medical maintenance or tapering of pharmacotherapy
Long-term sustained recovery
○ Major goal—continued primary responsibility for life ▪ Taper of pharmacotherapy—quarterly or biannual check-up from substance abuse treatment program ▪ Continuing pharmacotherapy—continued regular check-up with substance abuse treatment provider ▪ Continued engagement with peer-based recovery support program ▪ Patient becomes a peer recovery support for other patients
